# Generalized extragenital lichen sclerosus et atrophicus in skin of color

**DOI:** 10.1016/j.jdcr.2023.07.031

**Published:** 2023-08-19

**Authors:** Christina Jiang, Elnara Muradova, Jun Lu

**Affiliations:** a University of Connecticut School of Medicine, Farmington, Connecticut; b Department of Dermatology, University of Connecticut Health Center, Farmington, Connecticut

**Keywords:** extragenital lichen sclerosus, lichen sclerosus, lichen sclerosus et atrophicus

## Introduction

Lichen sclerosus et atrophicus (LSA) is a chronic inflammatory skin condition that typically affects the anogenital regions, but the extragenital variants comprise 15% to 20% of all LSA cases. Furthermore, only 6% of the LSA cases are of extragenital lichen sclerosus (EGLS) alone, without any genital involvement.^1^ Given the rare nature of extragenital-only forms of LSA, there are limited reports on this entity, with 1 case reported in the population with darker skin. Here, we reported a case series of generalized EGLS et atrophicus in 3 patients with Fitzpatrick Iv to Vskin and use of topical ruxolitinib as therapy in 1 patient.

## Case 1

**Fig 1:**
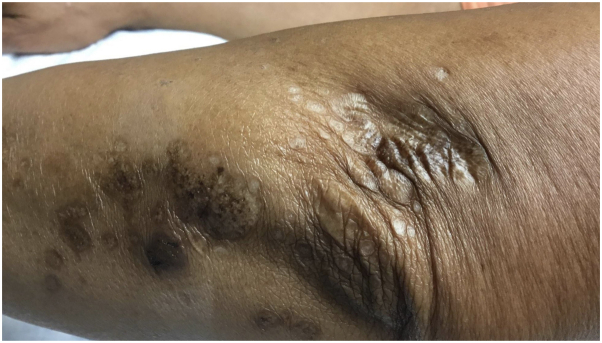
Well-demarcated hyperpigmented atrophic papules and plaques in a linear configuration on the left elbow.

**Fig 2:**
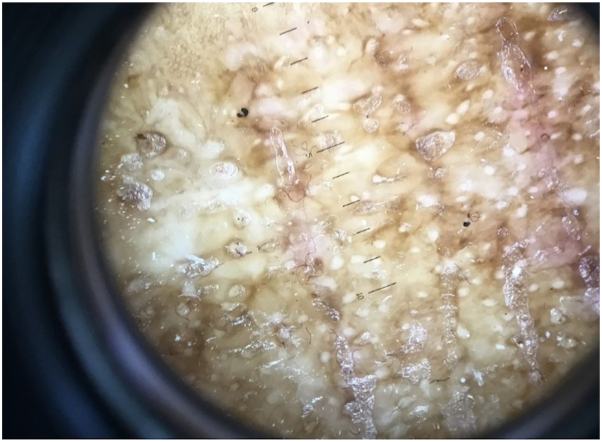
Follicular prominence with comedo-like openings was evident under dermatoscopy.

A 73-year-old African American woman presented to our dermatology clinic with a 2-year history of pruritic eruption, which began on the volar aspect of the wrists but spread to the trunk and extremities. Previous treatments included home remedies with cocoa butter and petrolatum jelly. She denied any bleeding, discharge, or ulceration. Physical examination revealed Fitzpatrick type V skin with widespread well-demarcated hypo- and hyperpigmented papules and plaques with parchment paper–like atrophy in a linear configuration involving wrists, arms, ankles, and upper portion of the back (Fig 1). Follicular prominence with comedo-like openings was evident under dermatoscopy (Fig 2). No genital involvement was observed. A punch biopsy of the left forearm was performed, and histology demonstrated epidermal atrophy with overlying orthohyperkeratosis, hyalinized papillary dermal collagen, perieccrine fat loss, and sparse perivascular and interstitial lymphocytic infiltrate. Based on the clinical and histopathological findings, a diagnosis of EGLS et atrophicus was made. She was treated with clobetasol 0.05% cream twice a day every other week for a total of 6 months, which led to improvement of pruritus.

### Case 2

**Fig 3:**
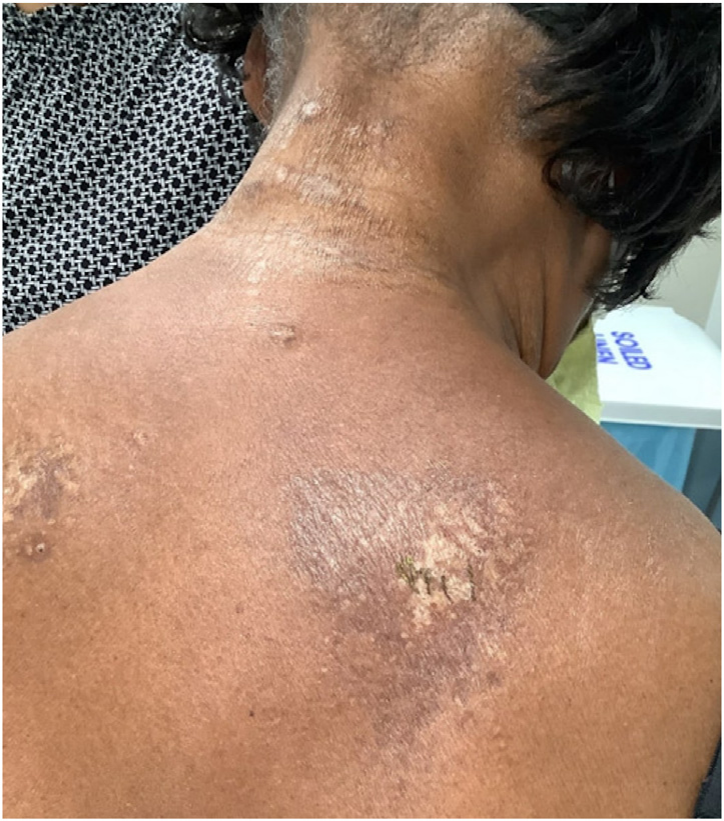
Well-demarcated hypo- and hyperpigmented plaques with yellowish atrophic patches on the posterior neck and upper portion of the back.

**Fig 4:**
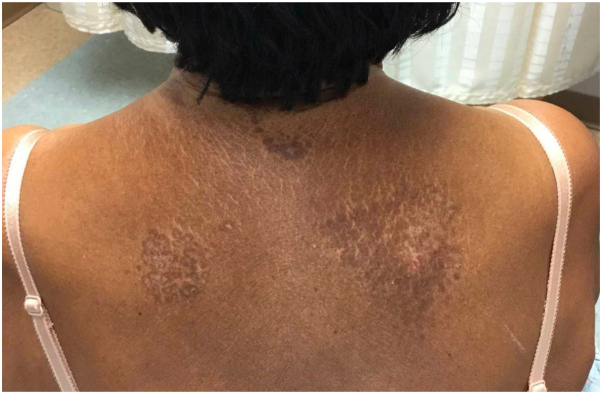
Postinflammatory hypo- and hyperpigmentation on the back 4 months after clobetasol therapy.

An 82-year-old Hispanic woman presented to our dermatology clinic for evaluation of a mildly pruritic rash of the neck, axilla, upper portion of the back, and chest for 1 year. This rash began shortly after suffering a myocardial infarction. The rash began on the neck but spread to her back, axillae, abdomen, and chest and gradually increased in size. The patient did not have any lesions in the genital region or oral cavity. Physical examination showed a Fitzpatrick type IV skin with well-demarcated hypo- and hyperpigmented plaques with yellowish atrophic patches on the anterior neck and right side of the upper portion of chest, upper portion of the back, posterior neck (Fig 3), abdomen, medial upper portion of the arm, and bilateral axillae. Skin punch biopsies of the midline and upper portion of the right chest demonstrated epidermal atrophy, focal basal vacuolization, pigmentary incontinence, hyalinization of papillary dermis, and follicular plugging, consistent with LSA. She was first treated with clobetasol 0.05% cream twice a day every other week for a total of 4 months, which led to improvement of pruritus and plaques with postinflammatory hypo- and hyperpigmentation (Fig 4).

## Case 3

**Fig 5:**
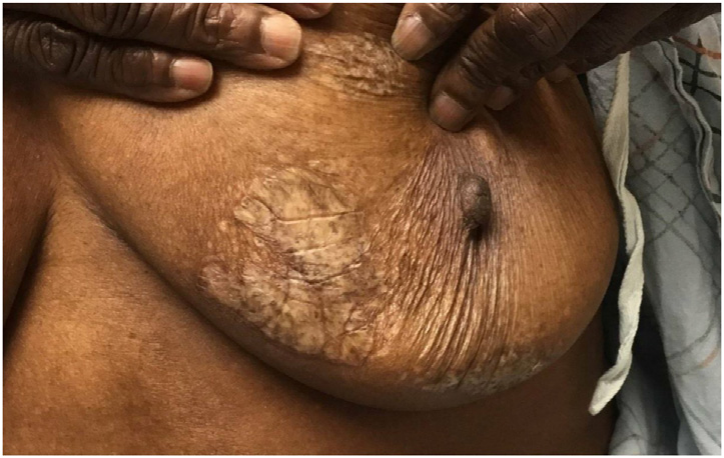
Hypopigmented patches on the left breast with skin atrophy in the center with scarring.

**Fig 6:**
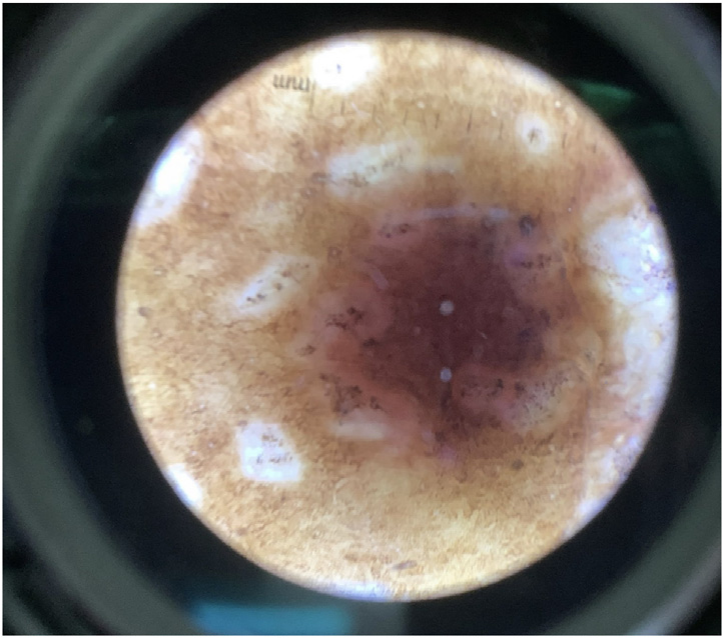
Dermatoscopy of the right breast showing white plaques with mottled pigmentation.

**Fig 7:**
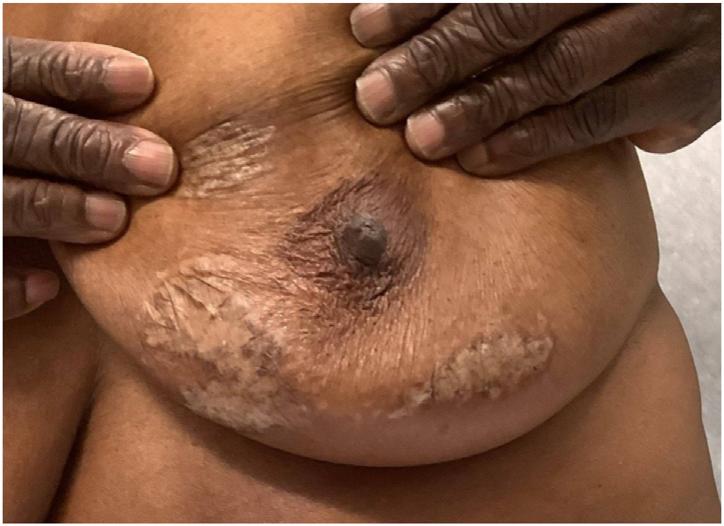
Decreased induration of the sclerotic plaques on the left breast after 2 months of topical 1.5% ruxolitinib therapy.

A 69-year-old woman with Fitzpatrick type V skin presented to our dermatology clinic for evaluation of a mildly pruritic rash on bilateral breasts and back that began 1 year ago, possibly triggered by an arthropod bite. Physical examination revealed hypopigmented patches on bilateral breasts with skin atrophy in the center along with scarring (Fig 5). There was no genital involvement. The lesions were slightly hyperkeratotic with surrounding hyperpigmentation with mottled pigmentation. Dermatoscopy of the right breast showed ivory plaques with comedo-like follicular plugging and mottled hypopigmentation (Fig 6). Skin biopsy demonstrated superficial dermal pallor associated with an interstitial lymphocytic and plasmacytic infiltrate suggestive of an overlap of lichen sclerosus (LS) and morphea. New lesions continued to develop on topical betamethasone and tacrolimus in the patient. She was then started on ruxolitinib 1.5% cream twice a day with significant improvement after 2 months, with less induration of the sclerotic plaques (Fig 7), and she reported markedly decreased pruritus and tenderness. We are planning on maintaining the patient on 1.5% cream for an additional 6 months for total treatment duration of 8 months.

## Discussion

LSA is a chronic inflammatory disease of unknown etiology, although hereditary, endocrine, infectious, and autoimmune factors are suggested to be involved. Antibodies to extracellular matrix protein-1 have been suggested to be involved in the mechanism of LS.^1^ LSA has been associated with multiple autoimmune diseases, including psoriasis, atopic dermatitis, systemic lupus, vitiligo, alopecia areata, and Graves’ disease, among others.^2^^,^^3^ LS can occur in both men and women but is more prevalent in women.^4^ Although the anal and genital regions are predominantly affected, EGLS comprises of 15% to 20% of all LS cases.^5^ Genital LS has a bimodal distribution, with 1 peak at puberty and another at menopause, whereas EGLS usually occurs in middle-aged adults.^2^ The most common symptoms are intractable pruritus and pain. The average age of patients with EGLS in this series is 75 years, which is significantly higher than that reported previously. All cases have Fitzpatrick skin type IV to V which has not been reported previously.

EGLS presents as asymptomatic white opalescent papules, patches, plaques, and erosions favoring the upper portion of the back, neck, and shoulders, and may become atrophic with time and appear parchment-like.^2^^,^^4^ The primary lesion begins as asymptomatic to mildly pruritic polygonal white papules that coalesce into well-demarcated erythematous plaques that become atrophic.^1^ Patients can frequently present with purpura.^2^ In patients with skin of color, EGLS can have various degrees of hypo- or hyperpigmentation.^4^

Diagnosis of EGLS can be made clinically and confirmed histopathologically. Dermatoscopy shows multiple patchy yellow-white structures, scales, keratotic plugs, pseudo-pigment-like pattern, and chrysalis structures.^2^ EGLS characteristics vary histologically based on the duration of the disease. Early disease is characterized by interface dermatitis with vacuolar/lichenoid changes.^2^ Progressive changes include follicular plugging, upper dermal sclerosis with a band of hyalinization, and inflammatory infiltrate. Late-stage disease is characterized by epidermal atrophy, loss of rete ridges, upper dermal sclerosis, and scanty inflammatory cellular infiltrates.^2^

The differential diagnoses include discoid lupus erythematosus, morphea, and hypopigmented mycosis fungoides.^2^, ^3^, ^4^ Hypopigmented mycosis fungoides presents clinically with multiple small hypopigmented or erythematous macules or patches on sun-exposed areas and may have associated pruritus.^6^ Histologically, there is infiltration of atypical lymphocytes in the epidermis and papillary dermis that express CD3 and CD4.^6^ A biopsy is required to confirm the diagnosis. Early indurated lesions of LS can appear like those of discoid lupus erythematosus clinically but could be easily differentiated through pathology.^6^ Clinically, early morphea often presents with erythematous plaques with violaceous halo, which is rarely seen in EGLS. Using dermatoscopy, follicular plugging can be easily identified in EGLS but not in morphea. Although both EGLS and morphea demonstrated epidermal atrophy, only EGLS has characteristic marked hyalinization of collagen with follicular plugging.^2^ Dermal sclerosis tends to be deeper in morphea with more perivascular infiltrations and mucin deposition. Therefore, clinicopathological correlation is important for differential diagnosis.

Notably, cases with coexistence of LS and morphea in the same lesions have been reported. An estimated 5% of patients with morphea have associated LS.^2^ The patient in case 3 has clinical presentation of typical EGLS with follicular plugging but histopathological features of both LS and morphea, suggesting that this patient may have overlap or coexistence of both entities.

Decision to treat lesions varies depending on the degree of patient’s symptoms, as EGLS is often minimally symptomatic or asymptomatic. First-line treatment for LS is ultrapotent topical corticosteroids.^5^ Topical calcineurin inhibitors such as tacrolimus and pimecrolimus are second-line topical agents but are not as effective as corticosteroids.^5^ All 3 patients were started on topical corticosteroids initially. The patient in case 2 improved with corticosteroids and was subsequently started on topical tacrolimus as maintenance therapy. The patient in case 3 did not respond adequately to either topical corticosteroids or tacrolimus; thus, she was started on topical ruxolitinib. For refractory cases, phototherapy has been used. Narrowband ultraviolet B radiation has been believed to delay the development of skin sclerosus by decreasing proinflammatory cytokines and increasing matrix metalloproteinases.^4^^,^^7^ Light therapy may be inconvenient in patients due to the scheduling and frequency of appointments; thus, we believed the best treatment options were topicals that can be applied at home. To our knowledge, herein, we reported the first case of EGLS responding to topical ruxolitinib, a new topical Janus kinase (JAK) inhibitor recently approved by the Food and Drug Administration to treat atopic dermatitis and vitiligo. The decision to start topical ruxolitinib was taken because the patient did not achieve treatment goal previously while receiving first-line topical corticosteroids and second-line topical calcineurin inhibitors. Long-term safety of topical ruxolitinib was evaluated in phase 3 studies for atopic dermatitis, in which non–melanoma skin cancers (NMSCs) were reported in 5 patients but were considered unrelated to the treatment. Furthermore, the NMSC in 4 of the 5 patients did not occur at the application site, which suggests the overall safety of topical application of JAK inhibitors.^8^ Although the risk of NMSC cannot be completely negated, it is important to continue screening patients for cutaneous malignancies. JAK is downstream of the TGF-beta mediated profibrotic signaling, and activation of the JAK/STAT pathway has been demonstrated to result in fibrosis,^9^ which is a sequela of EGLS and elucidates the potential mechanism of the use of topical ruxolitinib.^10^

EGLS is a chronic condition with residual scarring and pigmentary abnormalities.^4^ Compared with genital LS, EGLS has less carcinogenic potential; however, there have been a few reported cases with associated malignant transformations, particularly in cases of long-standing disease or in patients with genital involvement.^1^ Given this background, close follow-up of these patients is crucial.

In summary, we reported a case series of generalized EGLS et atrophicus without genital involvement, in the Fitzpatrick IV to V skin type. In addition, to our knowledge, this is the first report on successful use of topical ruxolitinib, a topical JAK inhibitor, to treat LSA. Further research is needed to understand the pathogenesis of this rare variant of LSA, especially in skin with a darker complexion. New generation of anti-inflammatory therapies, particularly JAK inhibitors, might be a promising therapy for LS or morphea.

## Conflicts of interest

None disclosed.
